# Promotion of In Vitro Hair Cell-like Cell Differentiation from Human Embryonic Stem Cells through the Regulation of Notch Signaling

**DOI:** 10.3390/metabo11120873

**Published:** 2021-12-15

**Authors:** Fengjiao Chen, Ying Yang, Jianling Chen, Zihua Tang, Qian Peng, Jinfu Wang, Jie Ding

**Affiliations:** 1 Key Laboratory of Plant Resource Conservation and Germplasm Innovation in Mountainous Region (Ministry of Education), Collaborative Innovation Center for Mountain Ecology & Agro-Bioengineering (CICMEAB), Institute of Agro-Bioengineering, College of Life Sciences, Guizhou University, Guiyang 550025, China; chenfengjiao0601@163.com (F.C.); yy2020021534@163.com (Y.Y.); 2 Institute of Cell and Development, College of Life Sciences, Zhejiang University, Hangzhou 310058, China; 20190004@lsu.edu.cn (J.C.); zihua.tang@thermofisher.com (Z.T.); 3 School of Medicine and Health Sciences, Lishui University, No. 1, Xueyuanlu Road, Lishui 323000, China; 4 Thermo Fisher Scientific, Block C, Global Trade Center, Dongcheng District, Beijing 100013, China; 5 Shanghai lnstitute of Nutrition and Health, University of Chinese Academy of Science, Shanghai 200031, China; pengqian2021@sibs.ac.cn

**Keywords:** human embryonic stem cells, progenitor cells, hair cell-like cells, Notch signaling pathway, shRNA

## Abstract

The Notch signaling pathway plays an important role in otic neurogenesis by regulating the differentiation of inner ear hair cells and supporting cells. Notch-regulated differentiation is required for the regeneration of hair cells in the inner ear. The temporal expression pattern of Notch ligands and receptors during in vitro hair cell-like cell differentiation from human embryonic stem cells (hESCs) was detected by quantitative reverse transcription-polymerase chain reaction (qRT-PCR). Subsequently, pAJ-U6-shRNA-CMV-Puro/GFP recombinant lentiviral vectors encoding short hairpin RNAs were used to silence JAG-1, JAG-2, and DLL-1, according to the temporal expression pattern of Notch ligands. Then, the effect of each ligand on the in vitro differentiation of hair cells was examined by RT-PCR, immunofluorescence, and scanning electron microscopy (SEM). The results showed that the individual deletion of JAG-2 or DLL-1 had no significant effect on the differentiation of hair cell-like cells. However, the simultaneous inhibition of both DLL-1 and JAG-2 increased the number of hair cell-like cells and decreased the number of supporting cells. JAG-2 and DLL-1 may have a synergistic role in in vitro hair cell differentiation.

## 1. Introduction

The Notch signaling pathway plays an important role in determining cell fate and function through proliferation, differentiation, and apoptosis as well as physiological and pathological processes, such as embryonic development, immunoregulation, tissue remodeling, and tumorigenesis [1,2,3]. Activation of the Notch signaling pathway results in the lateral inhibition of cellular differentiation through interactions between the extracellular domain of the Notch proteins and membrane-bound ligands (such as Delta, Serrate, and Jagged) on neighboring cells [4]. Thus, this inhibitory interaction with neighboring cells creates a mosaic cell pattern from initially homogenous cells. In addition, it has been verified that Notch signaling plays a number of roles in the development and regeneration of the inner ear in vertebrates [5,6,7]. Notch signaling mediates the committed induced differentiation of ear sensory cells, such as inner ear hair cells and supporting cells, from equivalent otic progenitor cells (OPCs), and promotes the formation of a precise arrangement of mosaics between hair cells and supporting cells [8,9]. NOTCH1 is an expression receptor at different stages of inner ear development, and Notch ligands involved in the differentiation of hair cells and supporting cells include Delta-like1 (DLL-1), Jagged1 (JAG-1), and Jagged2 (JAG-2) [10,11]. The differential spatio-temporal expression pattern of Notch receptors and ligands during inner ear development indicates that the activation of the Notch signaling pathway during different stages of development through distinct Notch ligands may have a diverse role in hair cell and supporting cell differentiation [10,12,13]. Activation of the Notch signaling pathway during the early development of the inner ear is propitious for the differentiation of otic progenitors. However, in the later development of the auditory epithelium, Notch signaling mediates alternate differentiation fates of equivalent OPCs through lateral inhibition. Specifically, OPCs with activated Notch signaling tend to differentiate into supporting cells, whereas OPCs with inhibited Notch signaling tend to differentiate into hair cells.

Embryonic stem cells (ESCs) are pluripotent stem cells that can differentiate into different cell lines through the ectoderm, mesoderm, and endoderm [14]. With respect to hair cell regeneration, it was reported that the differentiation of human embryonic stem cells (hESCs) induced in vitro produced hair cell-like cells and auditory neurons [15]. Under the induction of fibroblast growth factor (FGF), hESCs were differentiated into inner ear progenitor cells and expressed a variety of hair cell markers [16]. In addition, hESCs were induced to differentiate into hair cell-like cells with static cilia bundles [17]. As such, it is important to study the expression pattern of Notch receptors and ligands during in vitro differentiation of hair cells from ESCs, and the effects of regulating Notch ligand expression on the in vitro differentiation equilibrium of hair cells and supporting cells. Here, we examined the temporal expression pattern of Notch ligands and receptors during hair cell differentiation from hESCs. According to this temporal expression pattern, we constructed recombinant lentiviral shRNA vectors targeting JAG-1, JAG-2, and DLL-1. These lentivectors expressing JAG-1-, JAG-2-, and DLL-1-specific short hairpin RNAs (shRNAs) were transfected into the hESCs during different phases of in vitro hair cell-like cell differentiation. Subsequently, the effects of shRNA-mediated gene silencing (of Notch ligands) on the in vitro differentiation of hair cell-like cells were analyzed by RT-PCR, immunocytochemistry, and SEM. These findings could offer a theoretical foundation for research on the regulation of the in vitro differentiation equilibrium between hair cells and supporting cells derived from hESCs.

## 2. Results

### 2.1. Temporal Expression Pattern of Notch Ligands and Receptors during hESCs Hair Cell Differentiation

**Figure 1:**
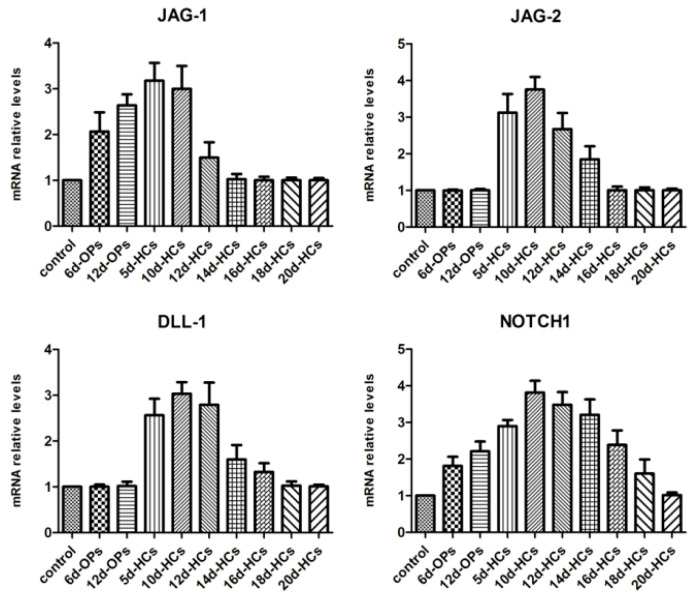
The expression pattern of Notch ligands and receptors. The expression of JAG-1 and NOTCH1 mRNA was gradually upregulated throughout the otic progenitor differentiation, whereas JAG-1 was downregulated during the mid-stage (day 14) and NOTCH1 was downregulated in the final stage (day 20) of hair cell differentiation. JAG-2 and DLL-1 mRNAs were detected at the early stage of hair cell differentiation, but their expression was downregulated by the mid-later stage of the differentiation. The statistical results were significant (*p* < 0.05). Control: undifferentiated hESCs; 6d-OPs/12d-OPs: cells on day 6/12 of otic progenitor differentiation; 5d-HCs/10d-HCs/12d-HCs/14d-HCs/16d-HCs/18d-HCs/20d-HCs: cells on day 5/10/12/14/16/18/20 of hair cell differentiation.

Examining the temporal expression pattern of Notch ligands and receptors during hair cell differentiation from hESCs was necessary to study the effects of Notch ligand gene silencing on the in vitro differentiation of hair cell-like cells. qRT-PCR was used to analyze the expression pattern of Notch ligands (JAG-1, JAG-2, and DLL-1) and receptor (NOTCH1) at the indicated times during the differentiation of hair cell-like cells from hESCs (Figure 1). The results indicated that the expression of JAG-1 mRNA was upregulated during the mid-stage of otic progenitor differentiation (from hESCs to OPCs) and downregulated during the mid-stage of hair cell differentiation (from OPCs to hair cell-like cells). The expression of JAG-2 was upregulated during the early stage of hair cell differentiation and downregulated during the mid-stage of hair cell differentiation. Expression of DLL-1 was initiated at the early stage of hair cell differentiation and ended at the mid-later stage. NOTCH1 mRNA was consistently detected throughout the differentiation of hESCs into hair cell-like cells and was finally downregulated during the final stage of hair cell differentiation. These expression patterns offered an important theoretical foundation for examining the effects of shRNA-mediated gene silencing of Notch ligands on the in vitro differentiation of hair cell-like cells.

### 2.2. The Effects of ShRNA Silencing on the Expression of Target Genes

Three series of lentiviral shRNA vectors (JAG-1-shRNA 1-4, JAG-2-shRNA 1-4, and DLL-1-shRNA 1-4) and one negative control vector (NC-shRNA) were constructed to determine the shRNA species with silencing efficiency for vectors. JAG-1-shRNA 1-4 vectors were used to infect cells on day 12 of otic progenitor differentiation, whereas JAG-2-shRNA 1-4 and DLL-1-shRNA 1-4 vectors were separately used to infect cells on day 5 of hair cell differentiation. The results of qRT-PCR showed that JAG-1, JAG-2, and DLL-1 in control (no transfection) and NC-shRNA transfected cells were expressed at the same levels (*p* > 0.05). The expression of JAG-1, JAG-2, and DLL-1 in cells transfected with JAG-1-shRNA 1-4, JAG-2-shRNA 1-4, and DLL-1-shRNA 1-4 vectors was downregulated relative to that of the control (*p* < 0.01), which had the highest silencing efficiency of their corresponding genes (Figure S1). Therefore, JAG-1-shRNA2, JAG-2-shRNA4, and DLL-1-shRNA3 vectors were chosen for subsequent stable infection.

After culturing in monolayers with otic progenitor-induction conditions for 10–12 days, hESCs can differentiate into OEPs and ONPs. The OEPs were separated, and hair cell differentiation was induced for 3–4 weeks. To examine the effects of Notch signaling on the in vitro differentiation of hair cell-like cells, lentivirus (pAJ-U6-shRNA-CMV-Puro/GFP) -expressing JAG-1-shRNA2, JAG-2-shRNA4, or DLL-1-shRNA3 was used to infect cells at different stages of differentiation. Six infection schemes were performed according to the aforementioned Notch ligand expression patterns, which included: J1-6d/J1-12d, designed such that cells on day 6/12 of otic progenitor differentiation were infected with JAG-1-shRNA2; J2-5d/D1-5d/J2+D1-5d, designed such that cells on day 5 of hair cell differentiation were infected with JAG-2-shRNA4/DLL-1-shRNA3/JAG-2-shRNA4 + DLL-1-shRNA3. After infection for 48h, the efficiency of GFP expression in each infection scheme was detected as shown in Figure S2.

After stably infected cells from each scheme were sorted by FACS, the GFP-expression of JAG-1, JAG-2, and DLL-1 was assessed by Western blotting. The results showed that the JAG-1 expression in cells of both the J1-6d and J1-12d schemes was significantly lower than that in control schemes (*p* < 0.05), and there was no significant difference between the two control schemes (*p* > 0.05; Figure S3A,B). In addition, JAG-2 and DLL-1 expressions in cells of the J2-5d, D1-5d, and J2+D1-5d schemes were significantly lower than that of both control schemes, and the infected efficiency of J2+D1-5d schemes was more obvious (*p* < 0.05; Figure S3C,D). These results confirmed the downregulation of JAG-1, JAG-2, and DLL-1 in the differentiating cells infected with the corresponding shRNAs.

### 2.3. The Expression of Early Otic-Specific Genes in OPCs Infected with JAG-1-shRNA2

**Figure 2:**
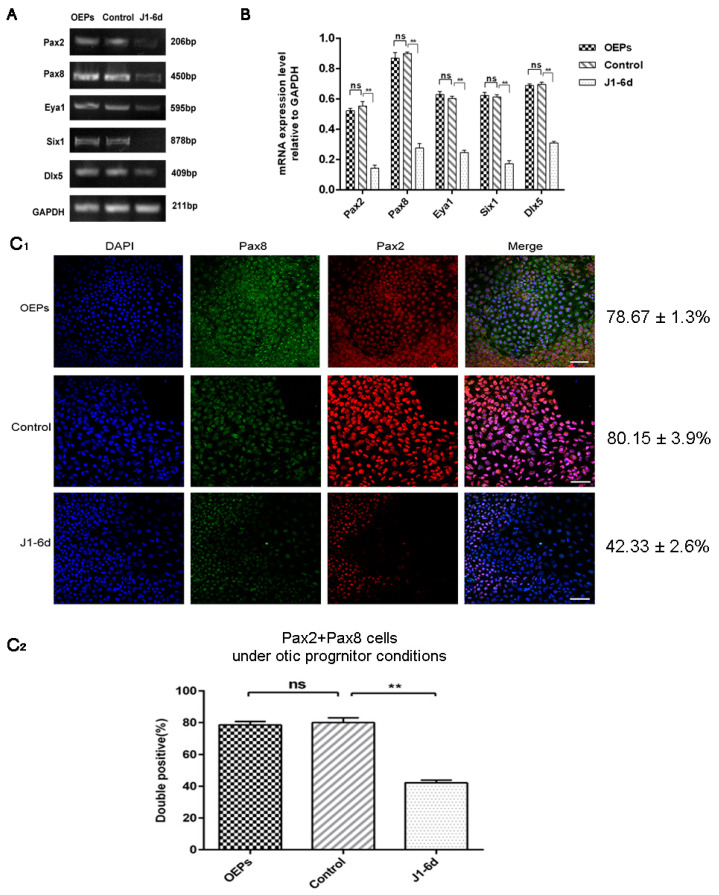
Analyses of early otic-specific markers in OPCs from the J1-6d infection scheme. (**A**) RT-PCR analyses for the expression of the early otic markers Pax2, Pax8, Dlx5, Six1, and Eya1. OEPs: no transfection; control: cells infected by lentivirus with NC-shRNA on day 6 of otic progenitor differentiation; J1-6d: cells infected with lentivirus harboring JAG-1-shRNA2 at day 6 of otic progenitor differentiation and then cultured for another 6 days under otic progenitor-inducing conditions. (**B**) Semi-quantitative analysis of the expression of specified genes by Image J and GraphPad. Expression values are relative to those of GAPDH and are presented as the mean ± SD (*n* = 3). ** indicates *p* < 0.01. (**C_1_**) Immunostaining of cells from the J1-6d infection scheme with antibodies specific for early otic markers. (**C_2_**) Co-expression of Pax2 and Pax8, specific for OPCs, in cells induced from the J1-6d infection scheme and control scheme. Error bars represent the SD (*n* = 5). The percentage of Pax8/Pax2 double-positive cells in the total cell population from the J1-6d infection scheme was significantly lower than that of the control scheme. Scale bar: 20 μm.

To study the function of JAG-1 in the in vitro differentiation of OPCs derived from hESCs, cells on day 6 of otic progenitor differentiation were infected with JAG-1-shRNA2 and cultured for another 6 days under otic progenitor-inducing conditions. As shown in Figure 2A,B, the specific expression levels of Pax2, Pax8, Eya1, Six1, and Dlx5 in cells from the J1-6d infection scheme were significantly lower than those in the cells infected with NC-shRNA, as shown by semi-quantitative RT-PCR (*p* < 0.01). Further, the percentage of Pax8/Pax2 double-positive cells in the J1-6d infection scheme was significantly lower than that of cells infected with NC-shRNA, as shown by immunostaining with specific antibodies (Figure 2C_1_,C_2_). Therefore, interference with JAG-1 could inhibit hESC differentiation into OPCs.

### 2.4. Analysis of Hair Cell-Specific Markers and Morphological Characteristics

**Figure 3:**
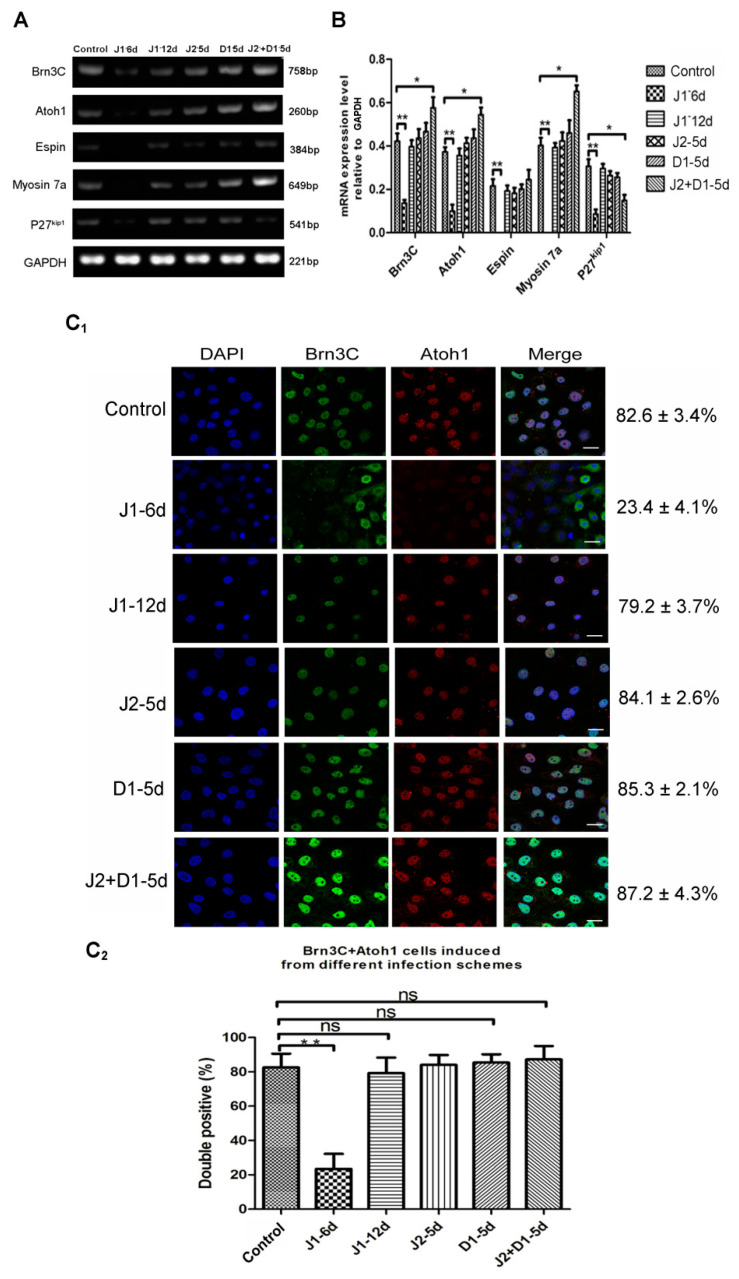

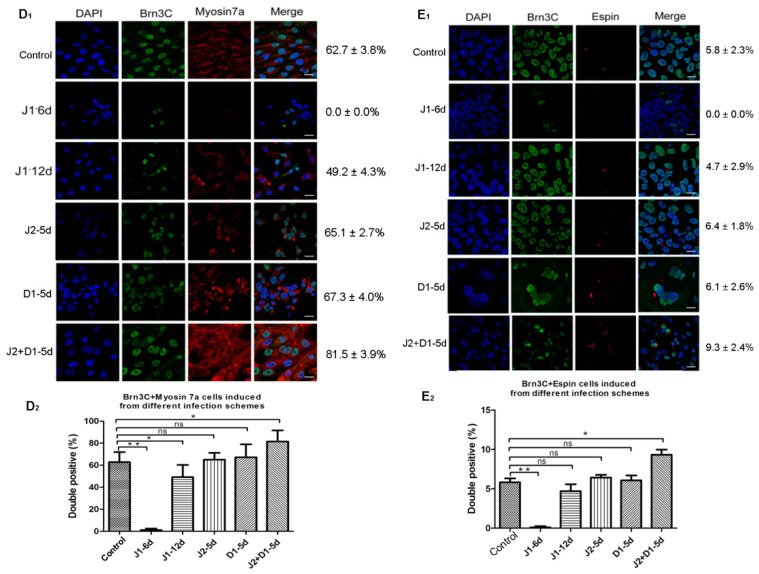
Analysis of hair cell- and supporting cell-specific markers in cells from six infection schemes. (**A**) RT-PCR analyses for the expression of markers specific for hair cells (Myosin7a, Brn3c, Atoh1, and Espin) and supporting cells (P27 ^kip1^) in cells from six infection schemes. (**B**) Semi-quantitative analysis of the expression of specific genes using Image J and GraphPad. Expression values are relative to those of GAPDH and are presented as the mean ± SD (*n* = 3). * indicates *p* < 0.05; ** indicates *p* < 0.01. (**C_1_**) Co-expression of Atoh1 (red) and Brn3c (green) in cells induced from six infection schemes. Scale bar: 20 μm. (**C_2_**) Percentage of Brn3C/Atoh1 double-positive cells in total cells induced using different infection schemes. Error bars represent the SD (*n* = 5). (**D_1_**) Co-expression of Myosin7a (red) and Brn3c (green) in cells induced using the six infection schemes. Scale bar: 20 μm. (**D_2_**) Co-expression of Brn3C and Myosin 7a in total cells induced using the different infection schemes. Error bars represent the SD (*n* = 5). (**E_1_**) Co-expression of Espin (red) and Brn3c (green) in cells induced using the six infection schemes. Scale bar: 20 μm. (**E_2_**) Percentage of Brn3C/Espin double-positive cells in total cells induced using the different infection schemes. Error bars represent the SD (*n* = 5). Control: cells infected by lentivirus with NC-shRNA on day 6 of otic progenitor differentiation; J1-6d/J1-12d: cells infected with lentivirus harboring JAG-1-shRNA2 at day 6/12 of otic progenitor differentiation and harvested for analysis at day 20 of hair cell differentiation; J2-5d/D1-5d/J2+D1-5d: cells infected with lentivirus harboring JAG-2-shRNA4/DLL-1-shRNA3/JAG-2-shRNA4 + DLL-1-shRNA3 at day 5 of hair cell differentiation and harvested for analysis at day 20 of hair cell differentiation.

To examine the effects of silencing various Notch ligands on hair cell differentiation from hESCs, total RNA was extracted from cells that were induced to undergo hair cell differentiation for 20 days, and was used to perform semi-quantitative RT-PCR. As shown in Figure 3A,B, in cells of the J1-6d infection scheme, the expression levels of hair cell- specific genes (Myosin7a, Brn3c, Atoh1, and Espin) or one supporting cell-specific gene (P27^kip1^) were all significantly lower than the cells infected with NC-shRNA (*p* < 0.01), while there was no significant difference in gene expression between cells from the J1-12d infection scheme and cells infected with NC-shRNA (*p* > 0.05) (Figure 3B). Accordingly, JAG-1 downregulation during otic progenitor differentiation not only inhibited hESC differentiation into OPCs, but also inhibited the differentiation and development of hair cells. However, interference with JAG-1 after the completion of otic progenitor differentiation had no significant effect on the differentiation of hair cell-like cells. The results demonstrate that JAG-1 primarily regulates the proliferation and differentiation of OPCs. In cells induced from the J2+D1-5d infection scheme, the expression of genes specific for hair cells was significantly higher than that of the cells infected with NC-shRNA, and the expression of P27^kip1^ was significantly lower than that of the cells infected with NC-shRNA (*p* < 0.05) (Figure 3B). Conversely, the J2-5d and D1-5d infection schemes had no significant interference effect (*p* > 0.05) (Figure 3B). These results showed that the downregulation of only one ligand (JAG-2 or DLL-1) could not significantly enhance the in vitro differentiation of hair cell-like cells, whereas interference with both ligands significantly upregulated the expression of hair cell-specific genes, demonstrating that JAG-2 and DLL-1 together play a role in the regulation of in vitro hair cell differentiation.

Subsequently, immunofluorescence staining was performed on cells induced to undergo hair cell differentiation for 20 days, using antibodies specific for Atoh1, Brn3c, and Espin (targeting proteins specific to hair cells) to analyze the effects of Notch ligands on in vitro hair cell differentiation. The results showed that all cells expressing Brn3c were immunolabeled by Atoh1. The rate of Brn3c/Atoh1 double-positive cells from the J1-6d infection scheme was significantly lower than that of the cells infected with NC-shRNA. There was no significant difference in the percentage of Brn3C-Atoh1 double-positive cells between any of the four infection schemes and the control scheme (Figure 3C_1_,C_2_). Moreover, the fluorescence intensities of Brn3c and Atoh1 in the Brn3c/Atoh1 double-positive cells from the J2+D1-5d infection scheme were much higher than the other four infection schemes. This was consistent with differences in Brn3c and Atoh1 expression among the five infection schemes, as shown in Figure 3B. Cells from the five infection schemes were also simultaneously labeled with antibodies specific for Brn3c and Myosin7a. As shown in Figure 3D_1_,D_2_, Myosin7a-positive cells were not detected in the J1-6d infection scheme. The percentage of cells co-expressing Brn3c and Myosin7a in the total cell population from the J1-12d infection scheme and the fluorescence intensity in some Myosin7a-positive cells were slightly lower than those of the cells infected with NC-shRNA. In addition, the fluorescence intensity of Myosin7a in some Myosin7a-positive cells from the J2+D1-5d infection scheme was significantly higher than that of the cells infected with NC-shRNA, while there was no significant difference in the positivity or fluorescence intensity between the other two infection schemes (J2-5d and D1-5d) and the control scheme. Then, cells from the five infection schemes were analyzed for the co-expression of Brn3c and Espin as shown in Figure 3E_1_,E_2_. Epsin was detected in cells from the control, J1-12d, J2-5d, D1-5d, and J2+D1-5d infection schemes, but was not detected in the J1-6d infection scheme. Espin was mainly distributed in bundles at one side of the positive cell, resembling stereociliary bundles. The rate of Brn3C/Espin double positive cells from the J2-5d and D1-5d infection schemes was not significantly different from that of the cells infected with NC-shRNA (Figure 3E_1_,E_2_). Only the percentage of Espin-positive cells in the J2+D1-5d infection scheme was higher than that of the cells infected with NC-shRNA (*p* < 0.05).

**Figure 4:**
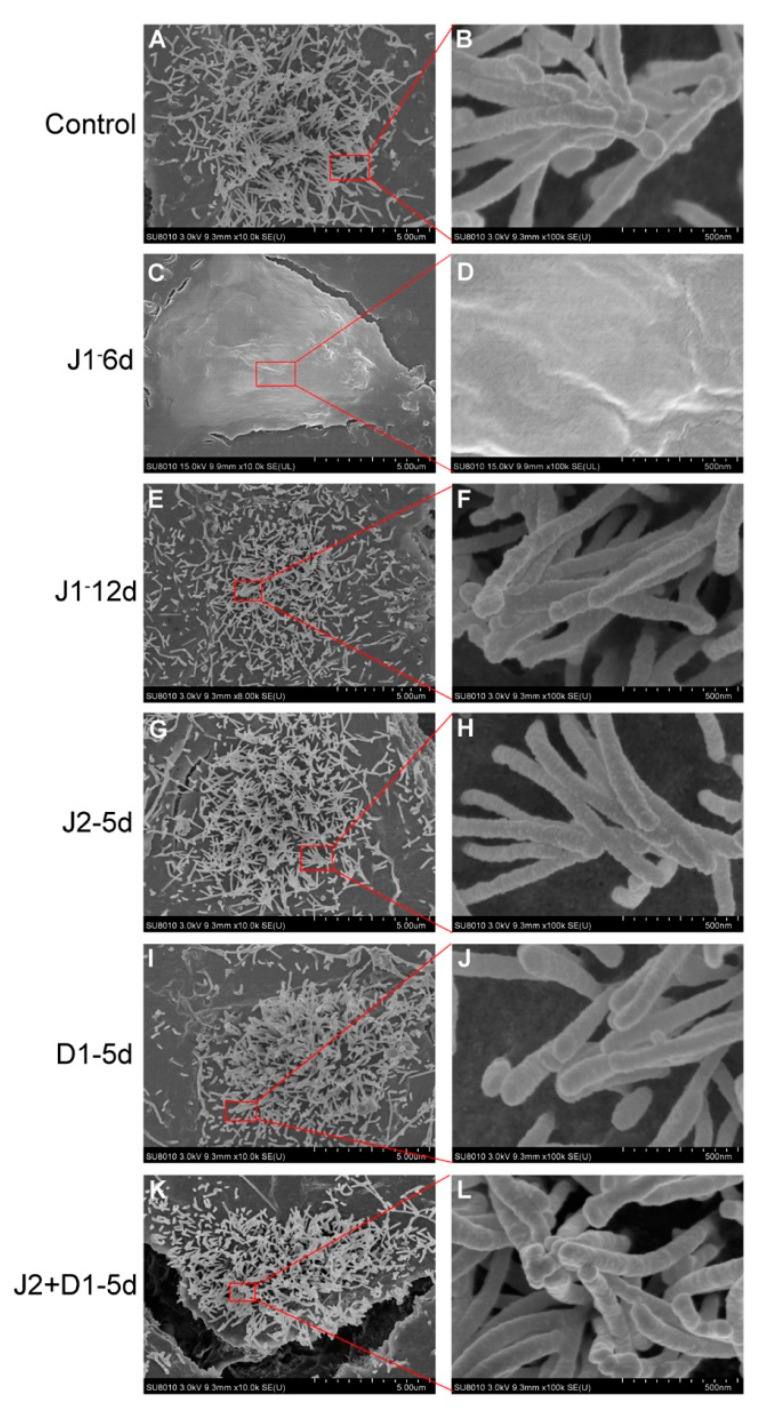
Scanning electron microscopy (SEM) analysis of hair cell-like cells from six infection schemes. (**A**,**B**) Stereocilia-like structures on the surface of hair cell-like cells induced from cells infected with NC-shRNA on day 6 of otic progenitor differentiation as the representative of all controls (Control). (**C**,**D**) No stereocilia-like structures were detected on the surface of hair cell-like cells induced using the J1-6d infection scheme. Stereocilia-like structures were detected on the surface of hair cell-like cells induced using the J1-12d (**E**,**F**), J2-5d (**G**,**H**), D1-5d (**I**,**J**), and J2+D1-5d (**K**,**L**) infection schemes.

Finally, SEM was selected to examine the structure and morphology of stereocilia on the surface of hair cell-like cells from the six infection schemes. As shown in Figure 4, cells from the J1-12d, J2-5d, D1-5d, and J2+D1-5d infection schemes had stereocilia-like structures and some stereocilia were bundled together with their tip-links (Figure 4E–L). This result was similar to that observed in the control cells (Figure 4A,B). However, stereocilia were not detected on the surface of cells from the J1-6d infection scheme (Figure 4C,D). Therefore, down regulation of JAG-1 not only suppressed otic progenitor differentiation, but also inhibited stereocilia-like structure formation on the surface of the induced cells.

## 3. Discussion

ShRNA, transduced by lentivirus, can be expressed stably in infected cell lines [18,19,20]. Therefore, lentivirus-harboring shRNA can be packaged, concentrated, and infected into the targeted cells to produce efficient and long-term gene silencing [21,22,23]. In this study, the constructed lentiviral shRNA vectors were transfected into target cells. The vectors with the highest interference efficiency to the three genes (JAG-1, JAG-2, and DLL-1) were selected by real-time PCR: JAG-1-shRNA2, JAG-2-shRNA4 and DLL-1-shRNA3 [24,25]. Subsequently, the proportion of GFP positive cells sorted by FACS showed that the infection efficiency on day 6 and 12 of otic progenitor differentiation was higher than that on day 5 of hair cell differentiation. This implies that OPCs should be easily infected by lentivirus. However, Western blotting to assess knockdown efficiency showed that the expression levels of targeted proteins in all infection schemes were significantly lower than that in the no transfection and NC-shRNA schemes, as expected.

The activation of the Notch signaling pathway promotes the formation of otic precursor cells during the early development of the inner ear. However, Notch signaling leads to two different cell fates in adjacent otic precursors through lateral inhibition during the subsequent development of the auditory sensory epithelium [7,26]. Otic precursors activated by Notch signaling differentiate into supporting cells, whereas those inhibited by Notch signaling differentiate into hair cells. Moreover, co-activation of cell cycle activator and Notch effectively transdifferentiated mature supporting cells into hair cells [27,28]. The prominent function of JAG-1 in Notch signaling is lateral induction, which is essential for the proliferation and development of OPCs and is necessary for hair cell differentiation [29,30,31,32,33,34]. Macchiarulo et al. found that knocking out JAG-1 in mouse ear canal vesicles led to failure of semicircular tube development and reduction of hair cells [35]. In the inner ear of the mouse, DLL-1 and JAG-2 inhibit differentiation into hair cells in adjacent OPCs and promote these cells to differentiate into supporting cells [36,37]. The simultaneous deletion of JAG-2 and DLL-1 leads to the loss of supporting cells as well as promotes the development of a large number of hair cells [38,39], which is consistent with the lateral inhibition hypothesis. In addition, the quantity of hair cells was not obviously changed when either JAG-2 or DLL-1 were individually deleted [6,10,40]. It has been speculated that JAG-2 and DLL-1 might play a synergistic role in the development of hair cells [10]. However, studies on the function of Notch signaling during the in vitro development and differentiation of hair cells and supporting cells from pluripotent stem cells are rare. Therefore, the expression pattern of Notch signaling during the in vivo differentiation of hair cells and supporting cells, as well as the effects of regulating Notch signaling on the in vivo differentiation of the two kinds of cells, require further investigation. In this study, JAG-1 was expressed in the mid-stage of otic progenitor differentiation. Therefore, downregulation of JAG-1 was induced in the cells at day 6 and 12 of otic progenitor differentiation (designated as J1-6d and J1-12d infection schemes, respectively). The expression of otic progenitor-specific genes and proteins in the J1-6d infection scheme was significantly lower than that of the control cells, which supported the previous statement “JAG-1 could stabilize prosensory fate” [34]. The GFP-positive cells in the J1-6d and J1-12d infection schemes were sorted by FACS and eventually induced to differentiate into hair cell-like cells. Subsequently, cells at day 20 of hair cell differentiation were analyzed by RT-PCR, immunocytochemistry, and SEM. The results showed that the expression of hair cell-specific genes and proteins in cells from the J1-6d infection scheme was significantly lower than that of the control cells, and no stereocilia-like structures were detected on the surface of these cells. Similarly, the expression of supporting cell-specific genes was significantly downregulated. These results verified that JAG-1 helped the progenitor cells with neurosensory function to differentiate into hair cells and supporting cells [41]. However, the genes and proteins expression of hair cells and supporting cells in the J1-12d infection protocol were slightly lower than that of the control. In addition, stereocilia-like structures could be detected. These results demonstrated that during the process of in vitro hair cell differentiation (from hESCs), downregulation of JAG-1 at the otic progenitor differentiation stage blocks the differentiation of OPCs, thus indirectly reducing the number of hair cell-like cells and supporting cells. The inhibition of JAG-1 expression in mature OPCs had no significant effect on the differentiation of hair cells and supporting cells. Therefore, we speculated that JAG-1 is involved in the differentiation of OPCs and promotes the differentiation and development of these cells.

During in vitro differentiation, JAG-2 and DLL-1 were detected in hair cells on day 5 of differentiation. Therefore, JAG-2 and DLL-2 were downregulated individually (J2-5d and D1-5d infection schemes) or simultaneously (J2+D1-5d infection scheme) in cells on day 5 of hair cell differentiation. Cells on day 20 of hair cell differentiation were analyzed by detecting the expression of genes or proteins and assessing stereociliary structure formation, which is specific to hair cells, to examine the role of JAG-2 and DLL-1 in the in vitro differentiation of hair cell-like cells. Downregulation of JAG-2 or DLL-1 in cells in the early stage of hair cell and supporting cell differentiation had no significant effect on the differentiation potential. In addition, stereocilia on the surface of cells derived from J2-5d, D1-5d, and J2+D1-5d infection schemes were well distributed, and we observed the existence of stereocilia-like structures with links between the tips of these structures on the surface of hair cell-like cells. This was similarly observed in cells of the control scheme. The expression of hair cell-specific genes and proteins was significantly higher when JAG-2 and DLL-1 were simultaneously downregulated, while the expression of supporting cell-specific genes was downregulated. Downregulation of only one of these ligands (JAG-2 or DLL-1) did not significantly increase the in vitro differentiation of hair cell-like cells; meanwhile, interference with both the ligands could significantly increase the differentiation of hair cells and promote the formation of stereocilia bundle-like structures on the surface of induced hair cells. This demonstrated that JAG-2 and DLL-1 might have a synergistic role in the regulation of hair cell-like cell differentiation. Similarly, ectopic proliferation of Sertoli cells and a reduced number of hair cells occurred in the cochlea with JAG-2/DLL-1 double mutations [36]. Apparently, these results emphasized that Notch-mediated lateral inhibition played an important coordinating role in the process of hair cell formation and supporting cell fate changes. Interestingly, in the absence of DLL-1, hair cells were early and overdeveloped, and the inner ear cell population was unbalanced; while JAG-1was silent, the total number of hair cells and supporting cells was significantly reduced. JAG-1 and DLL-1 mediated the inhibition of neighboring cells to generate the hair cell/supporting cell lattice through differential temporal expression. Nevertheless, this mode may be destroyed if the signal strength of JAG-1 and DLL-1 were stimulated at the same time [26,32,38]. These results could be explained as the unique but close relationship between multiple components of the Notch signaling pathway. Mutations or coordination in different components of the Notch signaling pathway may have varying degrees of impact on inner ear hair cells and supporting cells [42], which may have guiding significance for cell population development in hearing restoration therapy [43]. As such, coordination between different genes may promote the regeneration of hair cells, and the mechanism of regulating the differentiation of Notch signaling pathway in hair cells and supporting cells is worthy of further study. Undoubtedly, targeted gene therapy to help with hearing function recovery in people with sensorineural deafness will also become an attractive option for future research.

## 4. Materials and Methods

### 4.1. Two-Step Induction to Generate Hair Cell-like Cells from hESCs

The hESC line X1, obtained from Prof. Xiao, College of Animal Science, Zhejiang University, was cultured on inactivated mouse embryonic fibroblast (MEF) feeder cells with DMEM/F12 supplemented with 20% knockout serum replacement (KOSR; Gibco, Shanghai, China), 1% nonessential amino acids, 2 mM L-glutamine (Invitrogen, Shanghai, China), 0.1 mM 2-mercaptoethanol (Sigma, Shanghai, China), 50 mg/mL ampicillin, and 4 ng/mL of basic fibroblast growth factor (bFGF; Invitrogen). Before the induction of differentiation, the hESCs were dissociated into small clumps using collagenase IV (Invitrogen), and these small clumps were further dissociated using 0.025% trypsin-EDTA (Sigma). The tryptic digestion was terminated using a trypsin inhibitor, and the cell suspension was passed through a 100-μm cell strainer (BD Labware, Shanghai, China) to retain few clumps of two to three cells.

For otic progenitor differentiation, cells were plated at a density of 1 × 10^4^ cells/cm^2^ on laminin-coated plastic (5 μg/cm^2^; R&D systems, Shanghai, China) and incubated in DMEM/F12 supplemented with N2 (1:100), B27 (1:50), FGF3 (50 ng/mL), and FGF10 (50 ng/mL (Invitrogen) for 12 days. The medium was replaced with fresh medium every two days. After 12 days of differentiation, most hESCs had differentiated into otic progenitor cells. There were two morphologically distinct types of otic colonies [44]. One cell population exhibited a flat phenotype with a large amount of cytoplasm and formed epithelioid islands; these were identified as otic epithelial progenitors (OEPs). The second population had small cells with denser chromatin and presented with cytoplasmic projections; this population was identified as otic neural progenitors (ONPs). To enrich OEPs, cells surrounding the epithelial colony were lifted by a quick incubation with Accutase (Invitrogen) at 37 °C for 2–3 min. As the colony edges started to curl, cells surrounding the epithelial colonies were rinsed off. A prolonged Accutase treatment step permitted the collection of epithelial colonies that remained attached. For the induction of OEPs to differentiate into hair cell-like cells, we used laminin (Invitrogen) as a substrate [17]. Briefly, conditioned medium was used for hair cell differentiation. The conditioned medium was from a chicken utricle stromal cell culture and was supplemented with EGF (20 ng/mL) and RA (10^−6^ M). The conditioned medium was replaced every second day.

### 4.2. The Expression Pattern of Genes Specific for Notch Signaling Analyzed during the Different Stages of hESC Differentiation to Hair Cell-like Cells

Total RNA was extracted using Trizol reagent (TaKaRa, Shanghai, China) from cells in different stages of otic progenitor differentiation (otic progenitor differentiation for 6 and 12 days) and hair cell differentiation (hair cell differentiation for 5, 10, 12, 14, 16, 18, and 20 days). After reverse transcription, the expression at the mRNA level of Notch signaling pathway receptor NOTCH1 and ligands JAG1, JAG2, and DLL1 were analyzed by quantitative real-time polymerase chain reaction (qRT-PCR), which was performed with a LightCycler 480 using the default thermal cycling conditions (10 min at 95 °C and 45 cycles of 15 s at 95 °C plus 1 min at 60 °C). HPRT1 and B2M were used as the endogenous control genes for normalization. Relative quantification was performed using the comparative Ct (threshold cycle) method.

### 4.3. Recombinant Lentiviral Vector Construction and Cell Transfection

To target different regions of each mRNA sequence (Table S1), four shRNAs targeting four different sequences of JAG-1, JAG-2, and DLL-1 (JAG-1-shRNA 1-4 for JAG-1, JAG-2-shRNA 1-4 for JAG-2, and DLL-1-shRNA 1-4 for DLL-1) and one negative control vector (NC-shRNA) were constructed by Jikai Genetics (Shanghai, China) (Table S2). Each shRNA sequence was inserted into the lentiviral vector (pAJ-U6-CMV-Puro/GFP) to construct the recombinant lentiviral vector pAJ-U6-shRNA-CMV-Puro/GFP. Subsequently, three plasmids (pAJ-U6-shRNA-CMV-Puro/GFP, psPAX2 (gag/pol element), and pMD2.G (VSVG element)) were transfected into 293T cells using Lipofectamine 3000 to generate lenti-shRNA-GFP/puro viral particles. Reliable real-time PCR protocols were performed to titrate the lentivirus based on the number of proviral DNA copies present in the genomic DNA extracted from transduced cells or from vector RNA. These production and concentration methods resulted in high-titer vector preparations (Table 1).

Subsequently, JAG-1-recombinant lentiviral vectors were used to infect cells on day 12 of otic progenitor differentiation. JAG-2-recombinant lentiviral vectors or DLL-1-recombinant lentiviral vectors were used to infect cells on day 5 of hair cell differentiation. After 48 h, total RNA was extracted from these cells to perform qRT-PCR to analyze gene silencing efficiency. Then, the shRNA with the highest knockout efficiency was selected; we selected lentiviral vectors harboring these shRNAs to infect cells on day 6 and 12 of otic progenitor differentiation, and on day 5 of hair cell differentiation. After 48 h of infection, the cells were collected, filtered through a 300-mesh cell strainer, and suspended in 500 μL DMEM/F12 (minus phenol red) supplemented with 10 μM Y-27632, 200 U/ mL penicillin, and 200 µg/ mL streptomycin. GFP-expressing cells were then sorted by flow cytometry (FCM). To unify the timing of sorting, we started the differentiation procedure at different times to ensure the time consistency of infection, collection, and sorting for all conditions. The sorted cells infected on day 6 of otic progenitor differentiation were cultured under the required conditions for an additional 6 days. Subsequently, some cells were collected for the analysis of otic progenitor differentiation, and other cells were transferred to laminin-coated plastic containing conditioned medium supplemented with EGF (20 ng/mL), RA (10^−6^ M) and Y-27632 (10 μM) for 20 days of hair cell differentiation. The sorted cells infected on day 12 of otic progenitor differentiation and on day 5 of hair cell differentiation were directly transferred to laminin-coated plastic containing the conditioned medium supplemented with EGF (20 ng/mL), RA (10^−6^ M) and Y-27632 (10 μM) for 15 and 20 days of hair cell differentiation. The medium was replaced with fresh medium every second day. Cells infected with lentivirus containing NC-shRNA were used as a control for qRT-PCR analysis on the effects of each gene silencing on differentiation.

### 4.4. Western Blot

The cells were washed twice with ice-cold PBS and protein was extracted. The extract was centrifuged at 12,000 rpm for 15 min at 4 °C to remove cellular debris. Protein concentrations were determined by the Bradford method, where in 20 μg of protein sample was heated to 95 °C for 5 min, run on a 10% SDS-PAGE gel, and transferred to a PVDF membrane (Millipore, Shanghai, China) using the semidry transfer method. The membranes were blocked for 1 h in Tris-buffered saline containing 0.01% Tween 20 with 10% non-fat dried milk and incubated overnight at 4 °C with the relevant antibodies including anti-DLL-1, anti-JAG-1, and anti-JAG-2 (Santa Cruz Biotechnology, Shanghai, China). After washing, the membranes were incubated with a peroxidase-conjugated anti-IgG secondary antibody (Bio-Rad Laboratories, Shanghai, China) for 1 h. The protein bands were detected using an enhanced chemiluminescence detection kit. All the bands were analyzed using Image J software (version 1.6 NIH) to determine the relative levels of Notch signaling ligands compared to β-actin expression.

### 4.5. Gene Expression Specific Analysis

Total RNA was extracted and reverse transcribed (Fermentas, Shanghai, China). The cDNA was used as a template for PCR using the primer pairs listed in Table 2. All RT-PCR results presented were confirmed by at least two independent controlled experiments. PCR products were electrophoresed on a 1.2% agarose gel, and stained bands were visualized under UV light and photographed. Band intensities were analyzed quantitatively in triplicate using Image J software, and the expression level of each gene was normalized to that of GAPDH.

### 4.6. Immunocytochemistry

Cells were fixed with 4% paraformaldehyde for 15 min and permeabilized with PBS containing 0.25% Triton X-100 and 5% normal donkey serum for 10 min at room temperature. Blocking was performed in PBS containing 1% bovine serum albumin and 0.1% Tween-20, which was followed by three washes of 5 min each with PBS. The cells were incubated with primary antibody overnight at 4 °C. After washing, specific antibody binding was visualized with donkey anti-mouse, anti-goat, or anti-rabbit secondary antibodies conjugated to either Alexa Fluor 488 or Alexa Fluor 594 (Jackson, Shanghai, China). The dilution ratio of all antibodies was based on the reference [17]. Nuclei were visualized with DAPI (4′,6-diamidino-2-phenylindole). Images were acquired with a Zeiss Axiophot fluorescent and confocal microscope.

### 4.7. SEM Assay

Cells differentiated for 3 weeks were fixed overnight in 2.5% glutaraldehyde (Sigma, St. Louis, MO, USA) at 4 °C. The specimens were washed twice with PBS for 20 min each and treated with 1% osmium tetroxide (Sigma, St. Louis, MO, USA) for 30 min. The specimens were washed with PBS for 10 min and dehydrated in a graded ethanol series. Thereafter, ethanol was replaced with isoamyl acetate (Aladdin, Shanghai, China) for 20–30 min, and the specimens were dried using the critical point drying method. The specimens were viewed with a Hitachi S-3000N variable pressure SEM.

### 4.8. Statistical Analysis

Data are presented as mean values ± standard deviation (SD) with the number of independent experiments (n) indicated. All collected data were examined using a multifactorial analysis of variance. The statistical differences between the independent variables were assessed by post hoc tests (Tukey’s studentized range tests for variables). All tests were two-tailed, and statistical significance was set at *p* < 0.05.

## 5. Conclusions

A two-step induction protocol for generating hair cell-like cells from hESCs was used in this study. The first induction step is for the differentiation of hESCs into OPCs, whereas the second induction step is for the differentiation of OEPs into hair cell-like cells. Lentiviruses expressing JAG-1-, JAG-2-, and DLL-1-shRNA were used to infect cells in different phases of hair cell differentiation to silence specific Notch ligand genes. During the differentiation of OPCs, the downregulation of JAG-1 expression hindered otic progenitor differentiation and further resulted in insufficient differentiation into hair cell-like cells. The downregulation of JAG-1 expression in mature otic progenitors had no significant impact on hair cell and supporting cell differentiation. During the differentiation of hair cell-like cells, the downregulation of JAG-2 and DLL-1 could not significantly increase the differentiation potential of the cells. However, hair cell differentiation was significantly promoted and supporting cell differentiation was restricted when JAG-2 and DLL-1 were simultaneously downregulated. These results demonstrate that JAG-2 and DLL-1 may have a synergistic role in in vitro hair cell differentiation.

## Figures and Tables

**Table 1:** Analysis of lentivirus titers by quantitative PCR.

Lentivirus.	Item	V Value	C Value	N Value	D Value	Viral Titer	Mean Titer
JAG-1-shRNA2	1	10	37	1 × 10^5^	1	3.70 × 10^8^	
	2	1	3.98	1 × 10^5^	1	3.98 × 10^8^	3.70 × 10^8^
	3	0.1	0.34	1 × 10^5^	1	3.40 × 10^8^	
JAG-2-shRNA4	1	10	32	1 × 10^5^	1	3.20 × 10^8^	
	2	1	3.52	1 × 10^5^	1	3.52 × 10^8^	3.20 × 10^8^
	3	0.1	0.29	1 × 10^5^	1	2.90 × 10^8^	
DLL-1-shRNA3	1	10	27.6	1 × 10^5^	1	2.76 × 10^8^	
	2	1	3.05	1 × 10^5^	1	3.05 × 10^8^	2.74 × 10^8^
	3	0.1	0.24	1 × 10^5^	1	2.40 × 10^8^	
NC-shRNA	1	10	42	1 × 10^5^	1	4.20 × 10^8^	
	2	1	4.46	1 × 10^5^	1	4.46 × 10^8^	4.19 × 10^8^
	3	0.1	0.39	1 × 10^5^	1	3.90 × 10^8^	

**Table 2:** Primers of RT-PCR for marker genes specific for otic progenitors and hair cells.

Gene	Primer	Sequence	Tm
Pax8	Sense	5′- ACC CCC AAG GTG GTG GAG AAG A -3′	62 °C
	Antisense	5′- CTC GAG GTG GTG CTG GCT GAA G -3′	
Pax2	Sense	5′- GAG CGA GTT CTC CGG CAA C -3′	60 °C
	Antisense	5′- GTC AGA CGG GGA CGA TGT G -3′	
Six1	Sense	5′- GAC TCC GGT TTT CGC CTT TG -3′	57 °C
	Antisense	5′- TAG TTT GAG CTC CTG GCG TG -3′	
Dlx5	Sense	5′- TTC CAA GCT CCG TTC CAG AC -3′	57 °C
	Antisense	5′- GTA ATG CGG CCA GCT GAA AG -3′	
Eya-1	Sense	5′- TCA GAT GCT ATC TGC CGC TG -3′	57 °C
	Antisense	5′- GTG CCA TTG GGA GTC ATG GA -3′	
Atoh1	Sense	5′- GCC GCC CAG TAT TTG CTA CA -3′	57 °C
	Antisense	5′- GCT AGC CGT CTC TGC TTC TG -3′	
Myosin7A	Sense	5′- CAC ATC TTT GCC ATT GCT GAC -3′	55 °C
	Antisense	5′- AGA AGA GAA CCT CAC AGG CAT -3′	
Espin	Sense	5′- CAG GCA TGT CCT CAC CCA AT -3′	55 °C
	Antisense	5′- CGT GGC GGA GTT TGT TCT TG -3′	
Brn3c	Sense	5′- TGC AAG AAC CCA AAT TCT CC -3′	55 °C
	Antisense	5′- GAG CTC TGG CTT GCT GTT CT -3′	
P27^kip1^	Sense	5′- CTG GAG CGG ATG GAC GCC AGA C -3′	62 °C
	Antisense	5′- CGT CTG CTC CAC AGT GCC AGC -3′	
GAPDH	Sense	5′- GAA GGT CGG AGT CAA CGG -3′	58 °C
	Antisense	5′- GGA AGA TGG TGA TGG GAT T-3′	

## Data Availability

The data presented in this study are available in Supplementary Materials.

## Supplementary Materials

The following are available online at https://www.mdpi.com/article/10.3390/metabo11120873/s1, Table S1: Target information of shRNA sequence, Table S2: shRNA sequence, Figure S1: Efficiency of shRNA vectors, Figure S2: Infection efficiency of lentiviruses harboring shRNA, Figure S3: Identification of cells stably infected by the lentivirus.
