# Supplementary material for: Promotion of In Vitro Hair Cell-like Cell Differentiation from Human Embryonic Stem Cells through the Regulation of Notch Signaling
Source: Metabolites. 2021 Dec 15;11(12):873. doi: 10.3390/metabo11120873 (PMC8709284; doi:10.3390/metabo11120873)
Supplement: Supplementary file 1 [file metabolites-11-00873-s001.zip › metabolites-1497916-supplementary.pdf]

metabolites

(Supplemental material)

**Table S1.** Target information of shRNA sequence.

| ID | Sequence | Start | GC (%) |
|---|---|---|---|
| JAG1-RNAi(1) | GAATGGAGTACATCGTATA | 4049 | 36.84 |
| JAG1-RNAi(2) | GTTCAACCTGACAGTATTA | 847 | 36.84 |
| JAG1-RNAi(3) | GAGCACATTTGCAGTGAAT | 3340 | 42.11 |
| JAG1-RNAi(4) | AGGATAACTGTGCGAACAT | 3275 | 42.11 |
| JAG2-RNAi(1) | CTCACACAAATTCACCAAA | 3993 | 36.84 |
| JAG2-RNAi(2) | TCTACCAGTGCAAGAACTT | 3845 | 42.11 |
| JAG2-RNAi(3) | CTGCTACGACCTGGTCAAT | 2466 | 52.63 |
| JAG2-RNAi(4) | ACTGCCATATCAACGTCAA | 1772 | 42.11 |
| DLL1-RNAi(1) | ACGTCATATCCGAGGAGAA | 2439 | 47.37 |
| DLL1-RNAi(2) | CGGGCTGTTCAACTTCAAA | 2397 | 47.37 |
| DLL1-RNAi(3) | CCGACAAGAATGGCTTCAA | 2196 | 47.37 |
| DLL1-RNAi(4) | TGTTCTAATGGTGCCAAGT | 1556 | 42.11 |

Control: NC

Sequence: TTCTCCGAACGTGTCACGT

**Table S2.** shRNA sequence.

| ID | 5′ | Stem | Loop | Stem | 3′ |
|---|---|---|---|---|---|
| Jag-1-shRNA1-a | Ccgg | ccGAATGGAGTACATCGTATA | CTCGAG | TATACGATGTACTCCATTCGG | TTTTTg |
| Jag-1-shRNA1-b | aattcaaaaa | ccGAATGGAGTACATCGTATA | CTCGAG | TATACGATGTACTCCATTCGG | |
| Jag-1-shRNA2-a | Ccgg | ccGTTCAACCTGACAGTATTA | CTCGAG | TAATACTGTCAGGTTGAACGG | TTTTTg |
| Jag-1-shRNA2-b | aattcaaaaa | ccGTTCAACCTGACAGTATTA | CTCGAG | TAATACTGTCAGGTTGAACGG | |
| Jag-1-shRNA3-a | Ccgg | cgGAGCACATTTGCAGTGAAT | CTCGAG | ATTCACTGCAAATGTGCTCCG | TTTTTg |
| Jag-1-shRNA3-b | aattcaaaaa | cgGAGCACATTTGCAGTGAAT | CTCGAG | ATTCACTGCAAATGTGCTCCG | |
| Jag-1-shRNA4-a | Ccgg | ccAGGATAACTGTGCGAACAT | CTCGAG | ATGTTCGCACAGTTATCCTGG | TTTTTg |
| Jag-1-shRNA4-b | aattcaaaaa | ccAGGATAACTGTGCGAACAT | CTCGAG | ATGTTCGCACAGTTATCCTGG | |
| Jag-2-shRNA1-a | Ccgg | ctCTCACACAAATTCACCAAA | CTCGAG | TTTGGTGAATTTGTGTGAGAG | TTTTTg |
| Jag-2-shRNA1-b | aattcaaaaa | ctCTCACACAAATTCACCAAA | CTCGAG | TTTGGTGAATTTGTGTGAGAG | |
| Jag-2-shRNA2-a | Ccgg | gcTCTACCAGTGCAAGAACTT | CTCGAG | AAGTTCTTGCACTGGTAGAGC | TTTTTg |

| | | | | | |
|---|---|---|---|---|---|
| Jag-2-shRNA2-b | aattcaaaaa | gcTCTACCAGTGCAAGAACTT | CTCGAG | AAGTTCTTGCACTGGTAGAGC | |
| Jag-2-shRNA3-a | Ccgg | cgCTGCTACGACCTGGTCAAT | CTCGAG | ATTGACCAGGTCGTAGCAGCG | TTTTTg |
| Jag-2-shRNA3-b | aattcaaaaa | cgCTGCTACGACCTGGTCAAT | CTCGAG | ATTGACCAGGTCGTAGCAGCG | |
| Jag-2-shRNA4-a | Ccgg | caACTGCCATATCAACGTCAA | CTCGAG | TTGACGTTGATATGGCAGTTG | TTTTTg |
| Jag-2-shRNA4-b | aattcaaaaa | caACTGCCATATCAACGTCAA | CTCGAG | TTGACGTTGATATGGCAGTTG | |
| Dll-1 shRNA1-a | Ccgg | gtACGTCATATCCGAGGAGAA | CTCGAG | TTCTCCTCGGATATGACGTAC | TTTTTg |
| Dll-1 shRNA1-b | aattcaaaaa | gtACGTCATATCCGAGGAGAA | CTCGAG | TTCTCCTCGGATATGACGTAC | |
| Dll-1 shRNA2-a | Ccgg | ctCGGGCTGTTCAACTTCAAA | CTCGAG | TTTGAAGTTGAACAGCCCGAG | TTTTTg |
| Dll-1 shRNA2-b | aattcaaaaa | ctCGGGCTGTTCAACTTCAAA | CTCGAG | TTTGAAGTTGAACAGCCCGAG | |
| Dll-1 shRNA3-a | Ccgg | cgCCGACAAGAATGGCTTCAA | CTCGAG | TTGAAGCCATTCTTGTCGGCG | TTTTTg |
| Dll-1 shRNA3-b | aattcaaaaa | cgCCGACAAGAATGGCTTCAA | CTCGAG | TTGAAGCCATTCTTGTCGGCG | |
| Dll-1 shRNA4-a | Ccgg | ccTGTTCTAATGGTGCCAAGT | CTCGAG | ACTTGGCACCATTAGAACAGG | TTTTTg |
| Dll-1 shRNA4-b | aattcaaaaa | ccTGTTCTAATGGTGCCAAGT | CTCGAG | ACTTGGCACCATTAGAACAGG | |

**Figure S1**

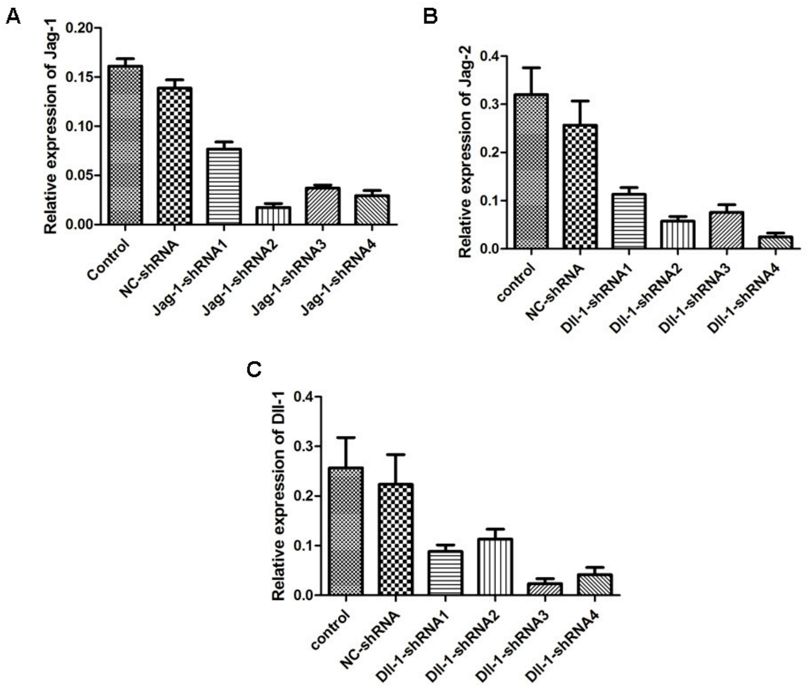


**Figure S1. Efficiency of shRNA vectors.** (**A**) JAG-1-shRNA: Cells on day 12 of otic progenitor differentiation were transfected with JAG-1-shRNA (1–4) and NC-shRNA. (**B**) JAG-2-shRNA: Cells on day 5 of hair cell differentiation were transfected with JAG-2-shRNA (1–4) and NC-shRNA. (**C**) DLL-1-shRNA: Cells on day 5 of hair cell differentiation were transfected with DLL-1-shRNA (1–4) and NC-shRNA. After 48 h of transfection, the total RNA was extracted and real-time PCR were performed to test the effects of various shRNAs on the expression of JAG-1, JAG-2, and DLL-1 respectively. Control: no transfection. The statistical results were significant ($p < 0.05$).

**Figure S2**

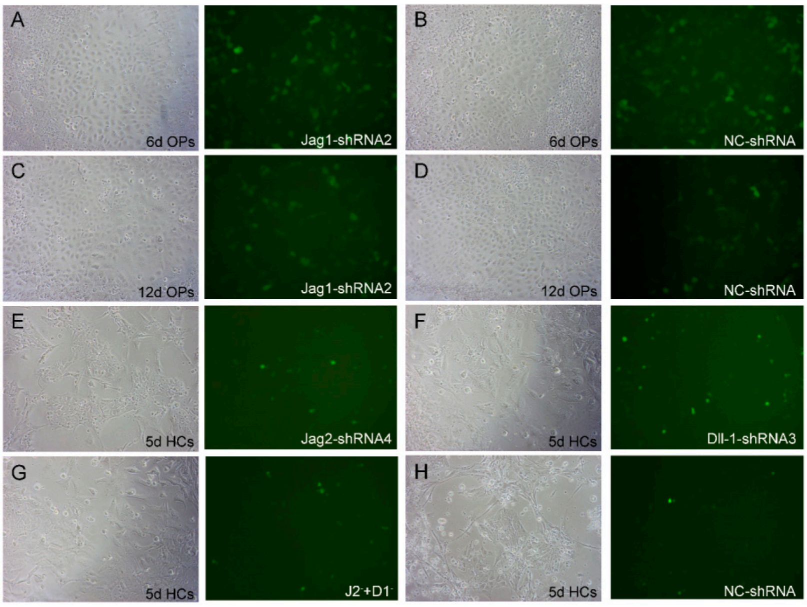


**Figure S2. Infection efficiency of lentiviruses harboring shRNA.** (**A**) Cell morphology and GFP expression in cells infected with lentivirus harboring JAG-1-shRNA2 on day 6 of otic progenitor differentiation. (**B**) Cell morphology and GFP expression in cells infected with lentivirus harboring NC-shRNA on day 6 of otic progenitor differentiation. (**C**) Cell morphology and GFP expression in cells infected with lentivirus harboring JAG-1-shRNA2 on day 12 of otic progenitor differentiation. (**D**) Cell morphology and GFP expression in cells infected with lentivirus harboring NC-shRNA on day 12 of otic progenitor differentiation. (**E**) Cell morphology and GFP expression in cells infected with lentivirus harboring JAG-2-shRNA4 on day 5 of hair cell differentiation. (**F**) Cell morphology and GFP expression in cells infected with lentivirus harboring DLL-1-shRNA3 on day 5 of hair cell differentiation. (**G**) Cell morphology and GFP expression in cells infected with lentiviruses harboring JAG-2-shRNA4 and DLL-1-shRNA3 on day 5 of hair cell differentiation. (**H**) Cell morphology and GFP expression in cells infected with lentivirus harboring NC-shRNA on day 5 of hair cell differentiation.

**Figure S3**

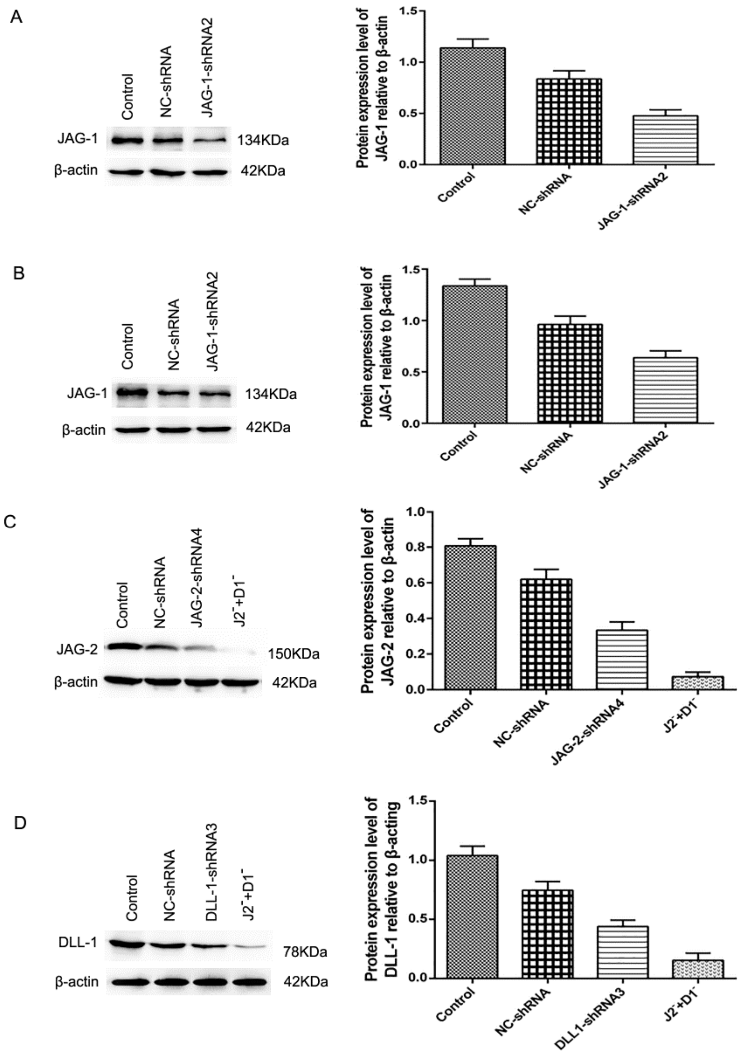


**Figure S3. Identification of cells stably infected by the lentivirus.** (**A**) and (**B**) Western blot analysis of cells infected with lentivirus harboring JAG-1-shRNA2 on day 6 and 12 of otic progenitor differentiation using an antibody specific for JAG-1. (**C**) and (**D**) Western blot analysis of cells infected with lentivirus harboring JAG-2-shRNA4 and DLL-1-shRNA3 at day 5 of hair cell differentiation using JAG-2 and DLL-1 specific antibodies.

Uninfected cells (Control) and cells infected with lentivirus harboring NC-shRNA were used as controls. The statistical results were significant ($p < 0.05$)
